# Induction of long-lasting protective immunity against *Toxoplasma gondii* in BALB/c mice by recombinant surface antigen 1 protein encapsulated in poly (lactide-co-glycolide) microparticles

**DOI:** 10.1186/1756-3305-6-34

**Published:** 2013-02-11

**Authors:** Shu-Chun Chuang, Jing-Chun Ko, Chaio-Ping Chen, Jia-Tze Du, Chung-Da Yang

**Affiliations:** 1 Department of Physiology, College of Medicine, Kaohsiung Medical University, No. 100, Shih-Chuan 1st Road, Kaohsiung, 807, Taiwan; 2 Graduate Institute of Animal Vaccine Technology, National Pingtung University of Science and Technology, No.1, Shuefu Road, Neipu, Pingtung, 912, Taiwan

**Keywords:** *Toxoplasma gondii* (*T. gondii*), Recombinant SAG1 (rSAG1), Poly (lactide-co-glycolide) (PLG), PLG-rSAG1 microparticles

## Abstract

**Background:**

Current development efforts of subunit vaccines against *Toxoplasma gondii*, the etiological agent of toxoplasmosis, have been focused mainly on tachyzoite surface antigen 1 (SAG1). Since microparticles made from poly (lactide-co-glycolide) (PLG) polymers have been developed as safe, potent adjuvants or delivery systems, we aimed to encapsulate recombinant SAG1 (rSAG1) with the PLG polymers to prepare PLG-encapsulated rSAG1 (PLG-rSAG1) microparticles that would sustain rSAG1 release and generate long-lasting protective immunity against *T. gondii* in BALB/c mice.

**Methods:**

In the present study, rSAG1 was encapsulated into PLG microparticles by the double emulsion method. PLG-rSAG1 microparticles were then intraperitoneally injected twice at a 14-day interval into BALB/c mice. We examined the ability of PLG-rSAG1 microparticles to induce and prolong effective anti-*Toxoplasma* immune responses, in comparison with rSAG1 formulated with a Vet L-10 adjuvant (rSAG1 (Vet L-10)). Eight weeks after the last immunization, protective activities were also evaluated after a lethal subcutaneous challenge of 1x10^4^ live *T. gondii* tachyzoites.

**Results:**

PLG-rSAG1 microparticles, 4.25~6.58 micrometers in diameter, showed 69%~81% entrapment efficiency. The amount of released rSAG1 protein from microparticles increased gradually over a 35-day period and the protein still retained native SAG1 antigenicity. Intraperitoneal vaccination of mice with the microparticles resulted in enhanced SAG1-specific IgG titers as well as lymphocyte proliferation and, more importantly, these enhanced activities were maintained for 10 weeks. In addition, eight weeks after the last immunization, maximum production of gamma interferon was detected in mice immunized with PLG-rSAG1 microparticles. Furthermore, 80% (8/10) of mice immunized with PLG-rSAG1 microparticles survived at least 28 days after a lethal subcutaneous tachyzoite challenge.

**Conclusions:**

Encapsulation of rSAG1 into PLG microparticles preserves the native SAG1 antigenicity and sustains the release of rSAG1 from microparticles. PLG-rSAG1 microparticles can effectively induce not only significant long-lasting SAG1-specific humoral and cell-mediated immune responses but also high protection against *T. gondii* tachyzoite infection. Our study provides a valuable basis for developing long-lasting vaccines against *T. gondii* for future use in humans and animals.

## Background

*Toxoplasma gondii*, the etiological agent of toxoplasmosis, is an intracellular protozoan parasite. *T. gondii* is widespread throughout the world and uses felines as final hosts and various endothermic animals, including humans, as intermediate hosts [1]. Toxoplasmosis imposes adverse economic impact due to the induction of severe abortion and neonatal loss of domestic animals [2]. In pregnant women, infection may give rise to serious fetal congenital mental retardation, blindness and hydrocephaly [3]. Toxoplasmosis is also a major opportunistic infection in immunocompromised individuals, often resulting in lethal toxoplasmic encephalitis [4].

Vaccines against *T. gondii* have been investigated for a long time. Although one attenuated vaccine has been successfully used to reduce abortions in sheep [5], it has a very short shelf-life and is unlikely to be used in humans [6]. In addition, many inactivated vaccines developed in the past have produced only little to moderate protective efficacy against infections with a lethal challenge dose of the virulent strain of *T. gondii*[7,8]. Current development efforts of subunit vaccines against the parasite have been focused mainly on the major immunodominant surface antigens of *T. gondii* tachyzoites [7], the rapidly multiplying stage during the acute phase infection. Among them, the surface antigen 1 (SAG1) has been identified to be involved in the process of host-cell invasion [9]. In addition, numerous studies have shown that vaccination with SAG1 in mice elicits a specific immune response and protection against *T. gondii* infection [6,7]. Therefore, the tachyzoite SAG1 can be considered as a possible candidate antigen for *Toxoplasma* vaccine development.

In our previous work, we cloned the *SAG1* sequence to produce a recombinant SAG1 (rSAG1) protein with a molecular weight of 30 kDa [10]. However, further protection analysis in mice demonstrated that rSAG1 emulsified with an oil adjuvant, Vet L-10, did not fully protect animals (60%) against a lethal subcutaneous challenge of *T. gondii* tachyzoites [10]. Thus, alternative potent adjuvants that can enhance the rSAG1 immunogenicity are needed to improve such moderate anti-*Toxoplasma* protection induced by the oil-formulated vaccine.

On the other hand, cell-mediated immunity is considered as the major mechanism in the prevention of *T. gondii* infection [7,11]. Th1-type cytokines, gamma interferon (IFN-γ) especially [12], secreted from CD4^+^ Th1 cells can subsequently activate CD8^+^ Tc cells to turn into major cytotoxic effector cells for lysing tachyzoite-infected cells, limiting parasite dissemination during the phase of acute infection [11] as well as inhibiting cyst formation during chronic infection [7]. These facts indicate that effective protection against *T. gondii* infection is critically dependent on the IFN-γ-associated Th1 cell-mediated immunity. Therefore, effective and trustworthy vaccines comprising subunit or recombinant antigens, such as rSAG1, formulated with potent adjuvants that are promised to induce an IFN-γ-associated Th1 cell-mediated immune response seem more likely to be approved for use.

In recent years, microparticles made from biodegradable and biocompatible polymers, such as poly (lactide-co-glycolide) (PLG), have been used as safe, potent adjuvants or delivery systems to encapsulate antigens for preparing controlled-release microparticle vaccines [13-15]. Adjuvant effects of PLG microencapsulation can protect antigens from unfavorable proteolytic degradation [15], allow the sustained and extended release of antigens over a long period [16], and facilitate antigen uptake via antigen-presenting cells (APCs) [15-18]. These effects in turn reinforce the antigen immunogenicity to favorably generate a strong immune response, especially Th1 cell-mediated immunity [13-15]. In other words, microparticle vaccines made from PLG polymers may fulfill the need for induction of a functional cell-mediated immune response against *T. gondii*. Although intranasal vaccination with one PLG microparticle vaccine containing a tachyzoite extract plus a mucosal adjuvant, cholera toxin, was described in sheep, the immune response produced was not sufficient to protect sheep against sporulated oocysts [19], indicating that other yet undefined factors are required.

In the present study, in order to enhance the rSAG1 immunogenicity, the PLG polymers were used as a potent adjuvant to encapsulate rSAG1 for preparing a controlled-release microparticle vaccine. The resulting PLG-encapsulated rSAG1 (PLG-rSAG1) microparticles were then injected intraperitoneally into BALB/c mice. We examined the ability of PLG-rSAG1 microparticles to induce and prolong effective anti-*Toxoplasma* immunity, in comparison with rSAG1 formulated with a Vet L-10 adjuvant (rSAG1 (Vet L-10)). Protective activities were also evaluated after a lethal subcutaneous challenge of *T. gondii* tachyzoites. We found that PLG encapsulation preserved the native SAG1 antigenicity, resulted in sustained release of rSAG1 for an extended period and, finally, allowed PLG-rSAG1 microparticles to induce and maintain humoral and cell-mediated immune responses against *T. gondii* in mice.

## Methods

### Mice and parasite antigens

Female ICR and BALB/c mice (6~8 weeks of age) were purchased from the National Laboratory Animal Center, National Science Council, Taiwan. In this study, ICR mice were used to maintain and passage *T. gondii* tachyzoites, while BALB/c mice were used in the immunization experiments. Mice were housed in high containment facilities and managed in compliance with the Animal Welfare Act. All administrations were reviewed and approved by The Institutional Animal Care and Use Committee, National Pingtung University of Science and Technology.

The tachyzoites of *T. gondii* (RH strain) used in this study were kindly provided by Dr. David Chao (Department of Biological Science, National Sun Yat-sen University, Kaohsiung, Taiwan) and maintained in ICR mice. The tachyzoites harvested from the peritoneal fluid of ICR mice infected intraperitoneally 2 days earlier with tachyzoites were washed three times with saline (150 mM NaCl), filtered through a 5-μm membrane (Millipore), and then concentrated by centrifugation at 2,000 × g for 10 min. Purified tachyzoites were then resuspended in a 10-fold volume of PBS (140 mM NaCl, 8.2 mM Na_2_HPO_4_, 1.5 mM KH_2_PO_4_, 2.7 mM KCl, pH 7.3), left at 4°C for 30 min, sonicated by using a VCX 130 ultrasonic processor (Sonics) equipped with a 3-mm diameter CV18 probe (30% of maximum power for four 10-sec pulses with 20-sec cooling between pulses), and then centrifuged at 12,000 × g for 30 min at 4°C. The resulting soluble supernatant was used as the tachyzoite sonicated antigen (TsoAg).

The rSAG1 protein used in the present study was produced according to the previous study [10]. The *SAG1* gene was re-cloned into the plasmid pGEX-6P-1 (GE Healthcare) and expressed as a glutathione-S-transferase (GST) fusion protein in BL21 (DE3) *Escherichia coli* (Yeastern Biotech). Briefly, SAG1 specific primers were designed (the forward primer: 5’-CCG*GAATTC*ATGTCGGTTTCGCTGCACCACTTCAT-3’ and the reverse primer: 5’-CGC*CCCGGG*CGCGACACAAGCTGCGATAGAGCC-3’ respectively contained the underlined *Eco*RI sequence as well as the underlined *Sma*I sequence) to carry out the SAG1 PCR amplification as before [10]. The amplified SAG1 fragment was digested with restriction enzymes *Eco*RI and *Sma*I (TOYOBO) and inserted into the *Eco*RI/*Sma*I sites of pGEX-6P-1, termed pGEX-SAG1. The recombinant plasmid was then transformed into BL21 (DE3) *E. coli*. After cloning, the induced fusion protein, GST-SAG1, was purified and its tag GST protein was removed as described previously [10]. The resulting recombinant protein, rSAG1 (30 kDa), was successfully prepared and its antigenicity was analyzed by Western blotting.

The protein concentrations of TsoAg and rSAG1 were determined by using the dye-binding DC protein assay (Bio-Rad) with bovine serum albumin (BSA) as a standard. Aliquots of these proteins were stored at −20°C until use.

### Monoclonal antibody (mAb)

The anti-SAG1 mouse mAb TG-1 (isotype G, subclass 1, κ light chain) used as a marker for SAG1 (30 kDa) in the present study was prepared as before [20], with minor modifications. BALB/c mice were subcutaneously injected twice at a two-week interval with TsoAg (50 μg) emulsified with Freund’s adjuvant (Sigma). Two weeks after the second immunization, each mouse was injected intravenously with 10 μg of TsoAg. One week later, spleen cells isolated from the immunized mice were fused with NS-1 myeloma cells (BCRC66036), which are sensitive to HAT (hypoxanthine-aminopterin-thymidine) (Sigma), in the presence of 50% polyethylene glycol (Sigma) for 1 min at 37°C and then cultured in 96-well culture plates with the HAT selection medium for one week. Wells with clones were screened for antibody production by enzyme-linked immunosorbent assay (ELISA). The hybridoma cells producing high anti-TsoAg titers were cloned by limiting dilution and then cultured for collecting mAb-containing supernatant media. The IgG fraction in the media was purified by the protein A agarose affinity column (Bio-Rad) and its specificity to SAG1 was determined by Western blotting. In addition, the mAb isotype was further determined by the IsoQuick™ isotyping kit (Sigma).

### Microparticle preparation

The rSAG1 protein was encapsulated in 50:50 poly (lactide-co-glycolide) (Sigma) using the water/oil/water double emulsion solvent evaporation technique as described previously [21,22], with minor modifications. Briefly, 10 ml of a 6% solution of PLG polymer in dichloromethane (Sigma) was mixed with 2 ml of a rSAG1 solution (5 mg/ml) using a PRO200 homogenizer (PRO Scientific) equipped with 10 mm × 150 mm generator at 12,000 rpm for 3 min to produce a water/oil emulsion. The resulting emulsion was further homogenized with 20 ml of a 2.5% polyvinyl alcohol (Sigma) solution at 15,000 rpm for 3 min to generate a stable water/oil/water emulsion. The water/oil/water emulsion was then stirred for 18 h at room temperature (RT) and pressurized to promote solvent evaporation and PLG-rSAG1 microparticle formation in a laboratory fume hood. The microparticles were collected by centrifugation at 4,000 × g for 30 min, washed three times with distilled water to remove non-entrapped rSAG1 and then lyophilized by a FD-5030 freeze dryer (Panchum) for storage at −20°C.

### Microparticle size and morphology

A total of 5 mg of freeze-dried PLG-rSAG1 microparticles was resuspended in 1 ml of deionized water in a 1.5 ml microfuge tube by vortexing. The particle size (diameter) was determined by N5 submicron particle size analyzer (Beckman Coulter). All measurements were performed in triplicate on samples from different batches. In addition, the particle morphology was inspected using scanning electron microscopy. The particle suspension was dropped onto stubs and allowed to air dry. After drying, the specimens were sputter-coated with gold and imaged with a S3000N scanning electron microscope (Hitachi).

### Protein entrapment in microparticles

A total of 5 mg of PLG-rSAG1 microparticles was first dissolved in 500 μl of 0.1 M NaOH with 2.5% SDS to extract the encapsulated rSAG1 as described previously [22]. After 4 h at 37°C, the extraction was terminated by adding 500 μl of 0.1 M HCl. After centrifugation at 12,000 × g for 10 min, the content of rSAG1 in the supernatant was assessed with the BCA protein assay (Pierce) and compared to BSA standards and adjusted against empty PLG microparticles. Based on this result, the ratio (w/w) of rSAG1 entrapped per dry weight of microparticles was determined and the entrapment efficiency (%) was expressed as a ratio of the actual rSAG1 entrapment to the theoretical rSAG1 entrapment by using the formula [22]: $\frac{\text{Actual}\;\text{rSAG}1\;\text{entrapment}\;\left(w/w\right)}{\text{Theoretical}\;\text{rSAG}1\;\text{entrapment}\;\left(w/w\right)}\times 100$. All measurements were performed in triplicate on samples from different batches.

### *In vitro* release study

A total of 5 mg of PLG-rSAG1 microparticles was suspended in 1 ml of PBS with 0.02% sodium azide and shaken at 37°C in 1.5 ml microfuge tubes. One milliliter of supernatant was sampled daily by centrifugation at 4,000 × g for 30 min and an additional 1 ml of fresh PBS was immediately added to the microfuge tubes in order to incubate as before [23]. The collected samples were neutralized and the amount of rSAG1 in the supernatant was measured using the BCA protein assay (Pierce), compared with BSA standards and adjusted against empty PLG microparticles. In addition, Western blot analysis using the mouse mAb TG-1, which is specific to SAG1 (30 kDa) of *T. gondii* tachyzoites, was performed to determine if released rSAG1 samples on days 1, 7, 14, 21, 28 and 35 exhibited the native SAG1 antigenicity. The released rSAG1 samples (500 μl) on days 1, 7, 14, 21, 28 and 35 were first concentrated 10-fold using the Amicon Ultra-0.5 Centrifugal Filter Device (10 kDa limit) (Millipore). The same volume (10 μl) of each concentrated rSAG1 sample was then separated by 12% SDS-PAGE and analyzed with mouse mAb TG-1 as described previously [10].

### Intraperitoneal immunization of mice

Five groups of 30 BALB/c mice each were intraperitoneally injected twice at a 14-day interval with PBS, blank PLG, 10 μg of soluble rSAG1 alone, 10 μg of rSAG1 emulsified with Vet L-10 (Invitrogen) oil adjuvant (rSAG1 (Vet L-10)) as described previously [10] or PLG-rSAG1 microparticles containing 10 μg of rSAG1. Specific anti-*Toxoplasma* immune responses were analyzed by the following immunoassays.

### Antigenic specificity of immunized sera

Two weeks after boosting, Western blot analysis was performed to further study the antigenic specificity of the immunized mouse sera. Briefly, aliquots of TsoAg (20 μg/well) were separated by 12% homologous SDS-PAGE and electrophoretically transferred to a polyvinylidene difluoride membrane (Millipore). After blocking, strips of the membrane were cut and probed with sera from mice immunized with PLG-rSAG1 microparticles, rSAG1 (Vet L-10), soluble rSAG1 alone, PLG or PBS for 1 h at 37°C. Incubation with mAb TG-1 was also conducted. IgG-bound antibodies on strips were detected with alkaline phosphatase-conjugated, 1:1,000-diluted goat anti-mouse IgG (Zymed) and the color development was then processed as described previously [10].

### Serum IgG titer assay

Following immunization, mouse sera were collected every two weeks and their IgG titers were measured by using ELISA as described previously [24], with minor modifications. Flat-bottomed 96-well polystyrene microplates (Nunc) were coated with 100 μl/well of TsoAg (10 μg/ml) in 0.1 M carbonate/bicarbonate buffer (pH 9.4) and incubated overnight at 4°C. Each well was then washed with PBS and blocked with the blocking buffer (PBS containing 5% BSA). Samples of 1:50 diluted serum in serial dilution were added to wells (50 μl/well) and incubated for 1.5 h at 37°C. After three washes with PBS-T (PBS with 0.05% Tween 20), wells were incubated with biotinylated goat anti-mouse IgG antibody (Zymed) diluted in the blocking buffer (1:3,000) for 1 h at 37°C. The plates were subsequently washed with PBS-T and streptavidin: peroxidase (1:3,000 dilution) was added. After incubation for 1 h at RT, plates were washed again with PBS-T and then incubated with 100 μl/well of tetramethylbenzidine substrate solution (Sigma) for 20 min in the dark. The enzymatic reaction was stopped with 100 μl/well of 1 M H_2_SO_4_ and the absorbance at 450 nm was read by an ELISA reader. The titer was defined as the reciprocal of the dilution that resulted in an absorbance value that is 50% of the total value, obtained by subtracting the background absorbance from maximum absorbance. The maximum absorbance is the absorbance at the plateau (around OD = 3.2~3.5) of the curve obtained by plotting the OD versus serial dilution of sera of immunized mice in a semi-logarithmical manner [24].

### Lymphocyte proliferation assay

Following immunization, three mice per group were sacrificed every two weeks to obtain spleen lymphocytes via gradient isolation by Ficoll-Paque™ Plus (GE Healthcare) under sterile conditions. The lymphocytes were then cultured in triplicate in 96-well culture plates at a concentration of 1 × 10^5^ cells per well in 200 μl of RPMI-1640 culture medium (CM). The cells in each well were stimulated with 10 μg/ml of TsoAg and incubated for 72 h at 37°C in 5% CO_2_. CM-treated cultures were also conducted to use as controls. The lymphocyte proliferation induced by TsoAg was monitored by using the BrdU (5-bromo-2’-deoxyuridine) Colorimetric Cell Proliferation ELISA (Roche) as described previously [10,20]. BrdU labeling solution (20 μl/well) was added into each well and incubated for an additional 12 h. Cells were then centrifuged at 2,000×g for 20 min and dried for 1 h at 60°C. Each well was fixed with 200 μl of the fixative solution for 30 min at RT. After washing, wells were incubated with the blocking reagent (200 μl/well) for 30 min at RT. After another wash, 100 μl of mouse anti-BrdU mAb conjugated peroxidase (1:100) was added to each well. After incubation for 1 h at 37°C, wells were washed and the substrate solution (100 μl/well) was added. The reaction was stopped 30 min later with 50 μl/well of 1 M H_2_SO_4_. The absorbance at 450 nm was measured. The stimulation index (SI = OD_450_ values from TsoAg-treated cultures/OD_450_ values from CM-treated control cultures) of each group was calculated as described previously [10,20] and expressed as the mean ± SD.

### IFN-γ assay

Eight weeks after boosting (before challenge), parallel triplicate lymphocyte cultures derived from three mice of each group were set up as per the procedure for proliferation assay. Cell cultures were stimulated with 10 μg/ml of TsoAg or 0.5 μg/ml of Con A (Sigma), a T cell mitogen, for 96 h at 37°C in 5% CO_2_. Cells stimulated with Con A were used as controls. Cell-free supernatants were harvested and their IFN-γ concentrations were analyzed by the sandwich ELISA using the OptEIA Mouse IFN-γ Set (BD Biosciences) according to the manufacturer’s instructions [10,20]. The concentrations of IFN-γ were determined by comparison to a standard curve created with known amounts of standard recombinant mouse IFN-γ and the sensitivity limit was 20 pg/ml.

### Tachyzoite challenge

Eight weeks after boosting, five groups of 10 mice each were challenged with a subcutaneous injection of 1 × 10^4^ live tachyzoites of *T. gondii* (RH strain) in order to verify whether the induced immune responses could protect mice from tachyzoite infection. Mice were observed daily for an additional 28 days and deaths were recorded as they occurred. The survival rate (number of surviving mice after challenge/number of tested mice in each group) in each group was calculated as described previously [10,20].

### Statistical analysis

Particle size and entrapment efficiency of microparticles from different batches, along with IFN-γ production from different immunization groups, were statistically compared using one-way ANOVA. Antibody titers were transformed logarithmically to attain normality. Log_10_ antibody titers and SI values of different immunization groups were statistically compared using the nested design. The survival rates of different groups were analyzed by the chi-square test [10,20]. A *P* value of less than 0.05 was considered a statistically significant difference.

## Results

### Antigenicity of purified *E. coli*-based rSAG1

**Figure 1:**
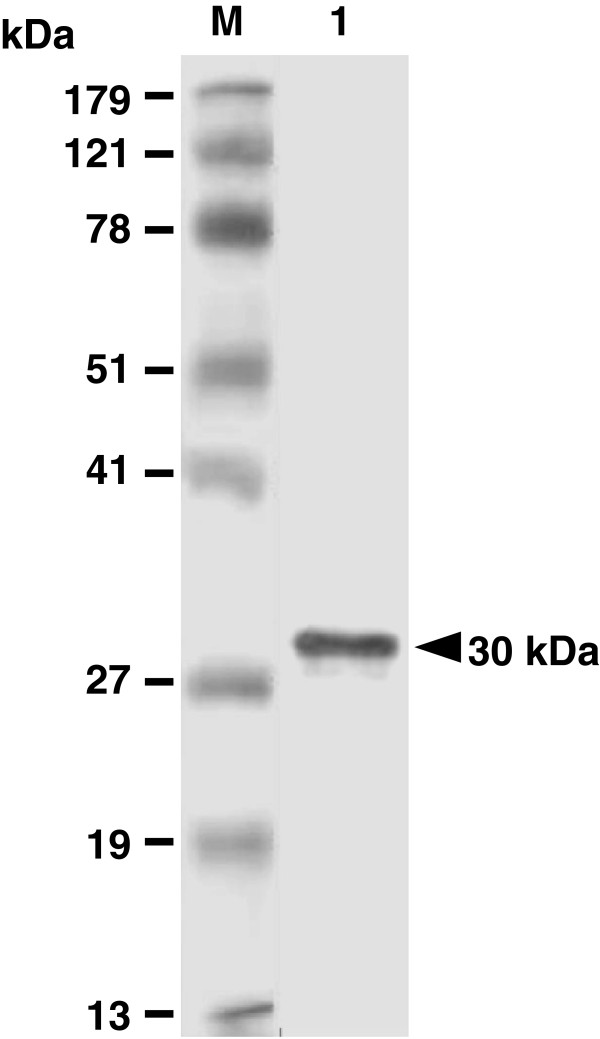
**Analysis of purified rSAG1 by Western blotting.** Purified rSAG1 was prepared as described in the Methods and analyzed with anti-SAG1 mouse mAb TG-1 (lane 1). Standard protein markers (lane M) are shown at the left.

After cloning, the induced GST-SAG1 protein was purified and its tag GST protein was removed. The resulting rSAG1 protein was analyzed by Western blot analysis using the mouse mAb TG-1, which is specific to SAG1 of *T. gondii* tachyzoites. The result demonstrated that rSAG1 protein (30 kDa) prepared in the present study showed the native SAG1 antigenicity recognized by the mouse mAb TG-1 (Figure 1).

### Characteristics of PLG-rSAG1 microparticles

**Figure 2:**
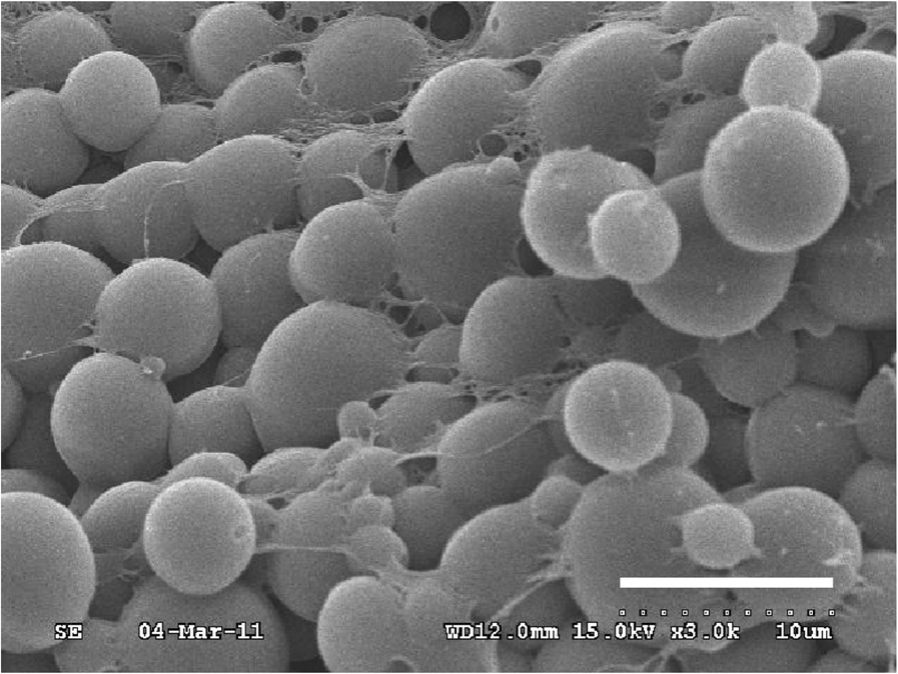
**Scanning electron micrograph of PLG-rSAG1 microparticles.** The morphology of PLG-rSAG1 microparticles was inspected by scanning electron microscopy and showed a uniform population of spherical particles with a smooth surface (bar represents 10 μm).

After PLG encapsulation, characteristics of PLG-rSAG1 microparticles were analyzed. The morphology of PLG-rSAG1 microparticles was inspected by scanning electron microscopy and showed a uniform population of spherical particles with a smooth surface (Figure 2). A particle size analyzer was further used to determine the particle size from 4.25 to 6.58 μm in diameter (Table 1). The entrapment efficiency for the rSAG1 protein ranged from 69% to 81%, without significant differences (*P>*0.05, ANOVA) among different batches (Table 1).

**Table 1:** Particle size and entrapment efficiency of PLG-rSAG1 microparticles

**Batch**	**Mean particle size (μm)**^**a**^	**Entrapment efficiency (%)**^**b**^
TPV −1	5.71±2.32^c^	75±12^d^
TPV −2	4.25±1.26^c^	69±10^d^
TPV −3	6.58±2.69^c^	81±15^d^

^a^ The particle size in diameter was measured and expressed as mean ± SD.

^b^ The entrapment efficiency was expressed as a ratio of the actual rSAG1 entrapment to the theoretical rSAG1 entrapment as described in Methods.

^c,d^A significant difference (*P*<0.05) exists between batches with different superscript letters.

### *In vitro* release of rSAG1 from microparticles

**Figure 3:**
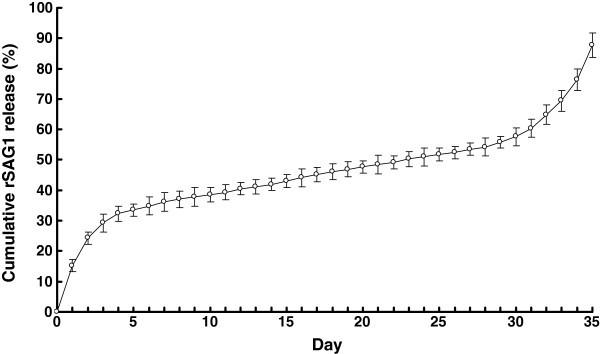
***In vitro *release of rSAG1 from PLG microparticles over a 35-day period.** A total of 5 mg of PLG-rSAG1 microparticles was suspended in 1 ml of PBS (pH 7.4) with 0.02% sodium azide and shaken at 37°C in 1.5 ml microfuge tubes for 35 days. The amount of rSAG1 in the supernatant sampled daily was measured using the BCA protein assay. The release studies were carried out in triplicate, with each point representing the mean ± SD.

The *in vitro* release of rSAG1 from PLG microparticles in PBS at 37°C was analyzed by the BCA protein assay (Figure 3). The cumulative rSAG1 release in the supernatant gradually increased over the course of a 35-day period with three distinct phases. Within the first three days, an initial burst released approximately 29.2% of the total protein load. Afterwards, there was a very slow release for 27 days followed by a rapid release during the last 5 days. Altogether, 87.8% of the total protein load was released from the microparticles during the 35-day study.

### Antigenicity of released rSAG1

**Figure 4:**
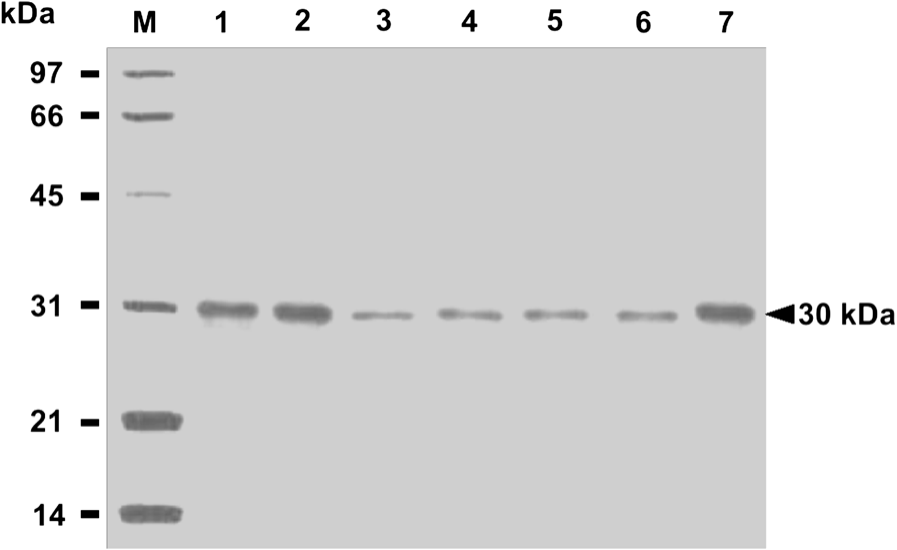
**Antigenicity of rSAG1 released from PLG microparticles.** The soluble rSAG1 (lane 1) and released rSAG1 samples on days 1 (lane 2), 7 (lane 3), 14 (lane 4), 21 (lane 5), 28 (lane 6) and 35 (lane 7) were analyzed by Western blotting with mouse mAb TG-1. Standard protein markers (lane M) are shown at the left.

To further determine if released rSAG1 from PLG microparticles retained native SAG1 antigenicity, Western blot analysis with use of mouse mAb TG-1, which is specific to the surface antigen SAG1 of *T. gondii* tachyzoites, was undertaken to examine released rSAG1 samples on days 1, 7, 14, 21, 28 and 35 (Figure 4). TG-1, which bound to the soluble rSAG1 protein (Figure 4, lane 1), recognized identical protein bands of 30 kDa displayed by the released rSAG1 proteins collected on days 1, 7, 14, 21, 28 and 35 (Figure 4, lanes 2~7). Thus, the rSAG1 protein retained the original SAG1 antigenicity following the encapsulation process and during the release from microparticles. In other words, the released rSAG1 from PLG microparticles prepared in our study had the potential to induce anti-SAG1 immunity.

### SAG1-specific serum response of immunized mice

**Figure 5:**
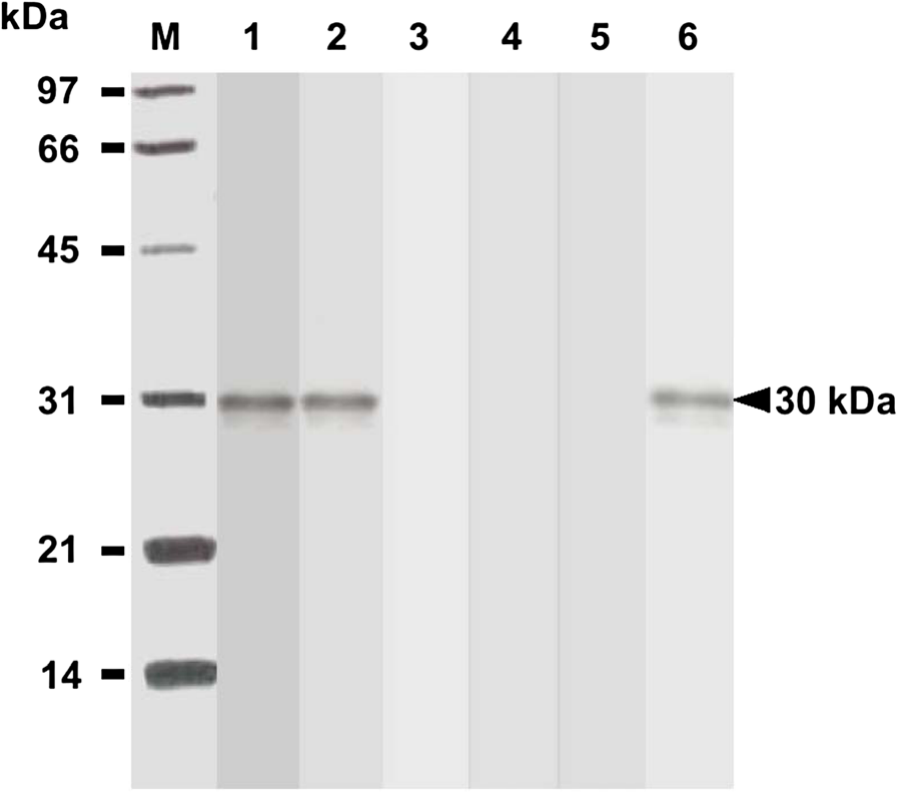
**Antigenic specificity of mouse sera.** Two weeks after boosting, mouse sera were collected to analyze their antigenic specificity. TsoAg was probed with sera from mice immunized with PLG-rSAG1 microparticles (lane 1), rSAG1 (Vet L-10) (lane 2), soluble rSAG1 alone (lane 3), PLG (lane 4) or PBS (lane 5). The mouse mAb TG-1 (lane 6) was conducted as a positive control for the native SAG1 in TsoAg. Standard protein markers (lane M) are shown at the left.

**Figure 6:**
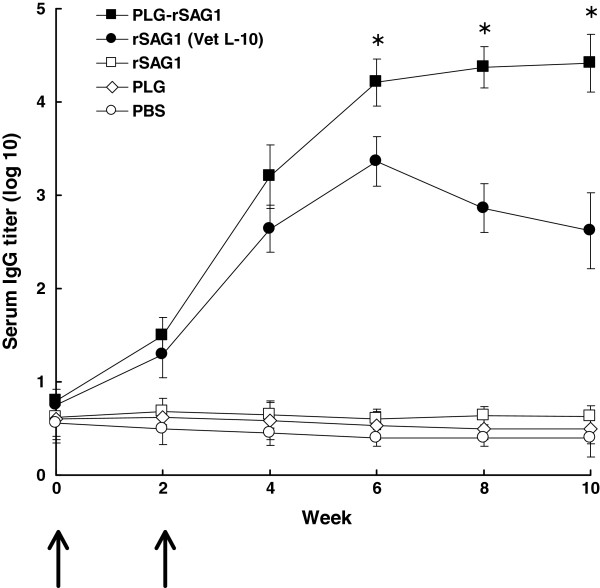
**IgG titers of mouse sera.** Groups of mice were intraperitoneally immunized twice (**↑**) with PLG-rSAG1 microparticles (■ ), rSAG1 (Vet L-10)(● ), soluble rSAG1 alone (□ ), PLG (◊ ), or PBS (○ ). Sera were collected from three mice per group every two weeks and their anti-*Toxoplasma* IgG titers were determined by ELISA. Results were presented as the mean of log_10_ titers ± SD. ^*^P<0.05 when comparing the PLG-rSAG1 group to the rSAG1 (Vet L-10) group.

The ability of PLG-rSAG1 microparticles to trigger humoral immunity against *T. gondii* in mice was subsequently evaluated. Western blot studies of sera obtained two weeks after boosting showed that both PLG-rSAG1 microparticles and oil formulation rSAG1 (Vet L-10) resulted in production of serum IgG antibodies against the native SAG1 protein in TsoAg (Figure 5, lanes 1 and 2), which was also recognized by the TG-1 mAb (Figure 5, lane 6). However, sera from mice immunized with soluble rSAG1 alone, PLG or PBS did not recognize anything in TsoAg (Figure 5, lanes 3~5). Therefore, intraperitoneal immunization with rSAG1 could elicit a specific serum response to the native SAG1 protein in TsoAg only when rSAG1 initially formulated with adjuvants, but not in its soluble form. These results were also consistent with those from Figure 4 and emphasized again that the released rSAG1 from PLG microparticles retained the native SAG1 antigenicity to induce anti-SAG1 immunity. In addition, the specific anti-*Toxoplasma* IgG titers of mouse sera, collected every two weeks, were determined by ELISA (Figure 6). Four weeks after boosting (the 6th week), IgG titers induced by PLG-rSAG1 microparticles were significantly higher (*P<*0.05, nested design) than those induced by rSAG1 (Vet L-10) and, more importantly, the high titers were maintained till the 10th week (Figure 6). Although high levels of antibodies were also induced by rSAG1 (Vet L-10) in the first six weeks, the levels gradually decreased starting from the 6th week to the 10th week (Figure 6). However, mice immunized with soluble rSAG1 alone, PLG or PBS displayed little, if any, anti-*Toxoplasma* IgG titers (Figure 6). Therefore, encapsulation of rSAG1 in PLG microparticles could elicit and prolong the high levels of anti-SAG1 antibodies, indicating the importance of use of the PLG adjuvant.

### SAG1-specific lymphocyte proliferation of immunized mice

**Figure 7:**
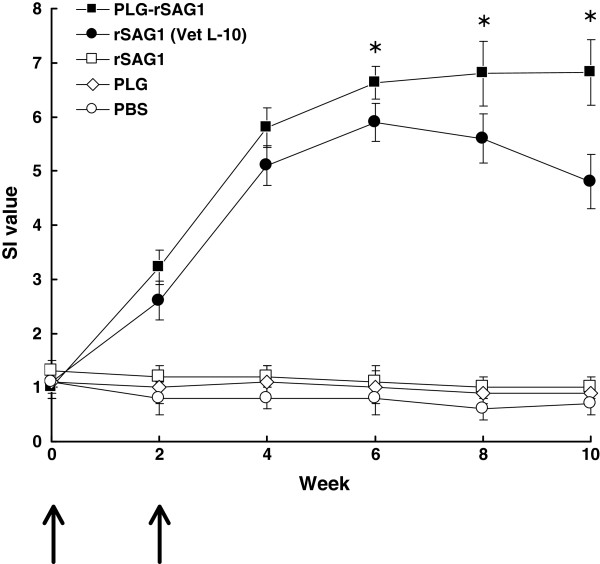
**Proliferation responses of mouse lymphocytes.** Groups of mice were intraperitoneally immunized twice (**↑**) with PLG-rSAG1 microparticles (■ ), rSAG1 (Vet L-10)(● ), soluble rSAG1 alone (□ ), PLG (◊ ), or PBS (○ ). After priming, TsoAg-stimulated spleen lymphocytes were prepared from three mice per group every two weeks and their subsequent proliferation responses were analyzed and expressed as stimulation index (SI) values. Results were presented as the mean of SI values ± SD. ^*^P<0.05 when comparing the PLG-rSAG1 group to the rSAG1 (Vet L-10) group.

After priming, TsoAg-stimulated spleen lymphocytes were prepared from mice of different groups every two weeks and their subsequent proliferation responses were analyzed and expressed as SI values (Figure 7). Four weeks after boosting (the 6th week), PLG-rSAG1 microparticles elicited significantly higher SI values (*P*<0.05, nested design) than rSAG1 (Vet L-10), and sustained high SI values till the 10th week (Figure 7). However, high SI values induced by rSAG1 (Vet L-10) during the first six weeks gradually decreased starting from the 6th week (Figure 7). Administration with soluble rSAG1 alone, PLG or PBS induced little lymphocyte proliferation in mice (Figure 7). Therefore, encapsulation of rSAG1 in PLG microparticles also rendered an enhanced and extended lymphocyte proliferation response to the native SAG1 protein in TsoAg.

### IFN-γ production of lymphocytes from immunized mice

To evaluate whether immunization with PLG-rSAG1 microparticles could induce a cell-mediated immune response in mice, eight weeks after boosting (the 10th week), the sandwich ELISA was used to analyze the ability of spleen lymphocytes to produce IFN-γ, a cytokine that plays an important role in cell-mediated immune responses. Upon TsoAg stimulation, lymphocytes from mice immunized with PLG-rSAG1 microparticles produced significantly higher levels of IFN-γ (*P*<0.05, ANOVA) than those from the rSAG1 (Vet L-10) group (Table 2). However, rSAG1-, PLG-, or PBS-administrated mice produced undetectable amounts of IFN-γ (<20 pg/ml). As a control, lymphocytes from all groups of mice were stimulated with T cell mitogen, Con A (0.5 μg/ml), and were found to produce similar amounts of IFN-γ (*P*>0.05, ANOVA). Therefore, the IFN-γ-associated cell-mediated immunity could be strongly elicited by immunization with PLG-rSAG1 microparticles.

**Table 2:** IFN-γ production by spleen lymphocyte cultures from immunized mice

**Group**	**IFN-γ (pg/ml)**^**a**^	
	**TsoAg**	**Con A**
PLG-rSAG1	1269 ± 193^b^	357 ± 45^d^
rSAG1 (Vet L-10)	545 ± 72^c^	362 ± 79^d^
rSAG1	< 20	353 ± 84^d^
PLG	< 20	392 ± 65^d^
PBS	< 20	348 ± 87^d^

^a^ The production of IFN-γ in the culture supernatants of TsoAg- or Con A-stimulated lymphocytes was calculated and expressed as mean ± SD.

^b,c,d^A significant difference (*P*<0.05) exists between groups with different superscript letters.

### Protection against *T. gondii* tachyzoite challenge

**Figure 8:**
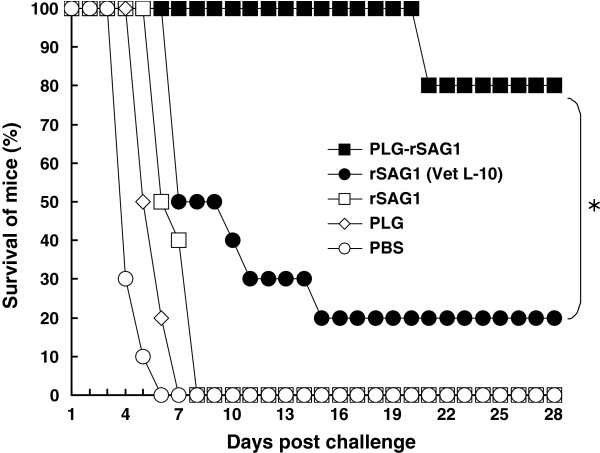
**Survival of immunized mice after a lethal tachyzoite challenge.** Groups of mice were intraperitoneally immunized twice with PLG-rSAG1 microparticles (■ ), rSAG1 (Vet L-10) (● ), soluble rSAG1 alone (□ ), PLG (◊ ), or PBS (○ ). Eight weeks after boosting, five groups of 10 mice each were subcutaneously infected with 1×10^4^ live tachyzoites of *T. gondii* (RH strain). Animals were observed daily for an additional month (28 days) and the final survival rates were calculated. ^*^P<0.05 when comparing the PLG-rSAG1 group to the rSAG1 (Vet L-10) group.

We then determined whether PLG-rSAG1 microparticles could confer effective protection in mice. Eight weeks after boosting (the 10th week), all groups of 10 mice each were subcutaneously challenged with 1 × 10^4^ live tachyzoites of *T. gondii* (RH strain). Animals were observed daily for an additional month (28 days) and the survival rates were recorded (Figure 8). All mice administrated with soluble rSAG1 alone, PLG or PBS died within 8 days after challenge and displayed no protection against the challenge. Two out of 10 mice immunized with rSAG1 (Vet L-10) survived during the challenge study and showed a low protection of 20%. However, only 2 mice died on the 21st day after challenge in the PLG-rSAG1-immunized group with the highest survival rate obtained in this group as 80%, which was significantly higher (*P<*0.05, chi-square test) than that of the rSAG1 (Vet L-10) group. Therefore, vaccination with rSAG1 encapsulated with the PLG polymers provided a substantial resistance to the experimental challenge of *T. gondii* tachyzoites.

## Discussion

Significant information obtained recently indicates that future investigations on *Toxoplasma* vaccine development will have to include adjuvants for enhancing the protective immune response against *T. gondii* in animals [6]. Different adjuvants capable of improving immune responses and protection against *T. gondii* have been described in numerous studies [7]. Among them, however, the biodegradable and biocompatible PLG polymers are so far rarely used as an adjuvant in the development of anti-*Toxoplasma* vaccines. To our knowledge, only one study proposed by Stanley and coauthors [19] has shown unexpected protection in sheep induced by a PLG microparticle vaccine containing a tachyzoite extract plus a mucosal adjuvant, cholera toxin. More effort is therefore needed to improve not only the stability of encapsulated *Toxoplasma* antigens but also the immune responses and protection they induce in animals.

In the present study, we have successfully encapsulated rSAG1 with PLG polymers and the resulting PLG-rSAG1 microparticles not only properly preserved the rSAG1’s antigenicity (Figure 4) but also sustained the controlled, stable release of the antigenic rSAG1 from PLG microparticles (Figure 3). Peritoneal immunization with the PLG-rSAG1 microparticles in mice further enhanced and maintained SAG1-specific immunity for an extended period (Figures 6 and 7) to protect mice from *T. gondii* tachyzoite infection (Figure 8). However, an immunity reduction starting from the 6th week to the 10th week in mice immunized with the oil formulation rSAG1 (Vet L-10) revealed that the adjuvant effect of Vet L-10 oil adjuvant is unable to maintain SAG1-specific immunity. Therefore, the ability of PLG-rSAG1 microparticles to control the release rate of antigenic rSAG1 is a particularly attractive characteristic.

In the present study, rSAG1 was encapsulated into PLG microparticles by the double emulsion method [22], which has been described to decrease contact between the antigen and the organic solvent containing the PLG polymer [21]. After PLG encapsulation, Western blot analysis with use of mouse mAb TG-1 further demonstrated that the released rSAG1 from microparticles during the 35-day release period still showed the native SAG1 antigenicity (Figure 4), which led to production of serum IgG antibodies against native SAG1 protein in TsoAg (Figure 5). Furthermore, the lack of major smaller or larger fragments of rSAG1 (as compared to the soluble rSAG1) revealed that very little denaturation or aggregation of the released rSAG1 occurred during the 35-day period (Figure 4). Even though we do not know if the rSAG1 released from microparticles was 100% intact, we reasonably believe that the antigenicity of released rSAG1 remained high enough since it induced subsequent immune responses (Figures 6 and 7) and protection (Figure 8). These data indicate that both the encapsulation procedure and release from microparticles in the present study are not detrimental to the antigenicity of rSAG1.

As *T. gondii* is an obligate intracellular parasite, protective immunity to *T. gondii* is largely mediated by Th1 cell-mediated immunity [7,11]. In addition, IFN-γ, one of Th1-type cytokines, has been demonstrated to be a decisive mediator of resistance to *T. gondii*[12]. Our previous studies [10,20] and that recorded by others [19] have shown that induction of both lymphocyte proliferation and high IFN-γ production positively correlates with protective Th1 cell-mediated immunity against *T. gondii*. In the present study, therefore, we concentrated much attention on the two activities of spleen lymphocytes from immunized mice and the results were used to judge whether protective Th1 cell-mediated immunity is induced. We found that an increased proliferation to the native SAG1 protein in TsoAg was readily observed in mice immunized with PLG-rSAG1 microparticles (Figure 7). More importantly, the enhanced proliferation response was extended till the 10th week (Figure 7). Eight weeks after boosting (the 10th week), maximum IFN-γ production was also detected in mice immunized with PLG-rSAG1 microparticles (Table 2). These findings indicate that immunization with PLG-rSAG1 microparticles really elicits the IFN-γ-associated Th1 cell-mediated immunity, which is required for prevention of *T. gondii* infection*.*

In addition, our results are consistent with those of previous studies, which have revealed that IFN-γ has to be secreted for as long as possible in order to maintain anti-*Toxoplasma* immunity [25,26]. The maximum IFN-γ production eight weeks after boosting (the 10th week) in mice immunized with PLG-rSAG1 microparticles (Table 2) could thus support the highest SAG1-specific proliferation response (Figure 7). However, the insufficient IFN-γ production in the rSAG1 (Vet L-10) group (Table 2) did not maintain a high proliferation response (Figure 7). These results further indicate that PLG encapsulation is better than oil emulsification in eliciting strong SAG1-specific IFN-γ production to maintain an anti-*Toxoplasma* immune response, indicating the importance of use of the PLG adjuvant.

According to previous studies, a strong cell-mediated immune response elicited by PLG microparticles appears to be largely a consequence of their uptake into antigen presenting cells (APCs) [15-18] and the delivery of these microparticle-containing APCs to specific lymphoid compartments [16,18] following vaccination. The particle size used for vaccination in animals is an important parameter in enhancing APC uptake [15]. Particles smaller than 10 μm in diameter are appropriate for direct uptake by APCs [17], such as macrophages and dendritic cells, that subsequently traffic to specific lymphoid compartments [16,18], thereby initiating a specific immune response [13-15]. The mean diameter of PLG-rSAG1 microparticles from different batches in the present study is smaller than 10 μm (4.25~6.58 μm as indicated by Table 1). The proper size range thus could stimulate peritoneal macrophages to facilitate the uptake of PLG-rSAG1 microparticles administered in the mouse peritoneal cavity. The microparticle-containing macrophages then traveled to other lymphoid compartments, including the spleen, and effectively presented SAG1 epitopes to T lymphocytes, especially Th1 and Tc, thereby inducing strong SAG1-specific Th1 cell-mediated immunity as shown in Figure 7 and Table 2. Therefore, facilitation of uptake and delivery of PLG-rSAG1 microparticles by macrophages can lead to more effective antigen processing and presentation to T lymphocytes capable of inducting cell-mediated immune responses [27,28]. However, the oil formulation rSAG1 (Vet L-10) used in this study could not result in more effective rSAG1 uptake and delivery than those induced by PLG-rSAG1 microparticles, and thus caused a weak Th1 cell-mediated immunity (low proliferation and IFN-γ production).

More importantly, the immune responses induced by PLG-rSAG1 microparticles protected mice (80%) against a lethal subcutaneous challenge of 1 × 10^4^*T. gondii* tachyzoites (RH strain) (Figure 8), an amount 10 times higher than that we had previously determined to be optimal for use as a challenge dose at 10 LD_50_[20], and enabled mice to survive for a long period of 28 days after challenge. Thus, the high survival rate and long survival period in mice encouraged us to believe that PLG-rSAG1 microparticles strongly induce a protective immune response (Th1 cell-mediated immunity) to eliminate tachyzoite-infected cells and to subsequently limit parasite dissemination during the experimental tachyzoite challenge [11].

In addition to cell-mediated immunity, an enhanced and extended anti-SAG1 IgG response detected in mouse serum following immunization with PLG-rSAG1 microparticles (Figures 5 and 6) also indicates that systemic humoral immunity mediated by B cells may participate in the resistance to *T. gondii* infection. Although the role of IgG antibodies in anti-*Toxoplasma* immunity is unclear, a general assumption has been proposed that serum IgG antibodies play a partial role in the prevention of secondary *T. gondii* infection [29,30].

Based on the results in the present study, peritoneal immunization with the SAG1 microparticle vaccine in BALB/c mice can induce not only significant long-lasting SAG1-specific humoral and cell-mediated immune responses but also high protection against *T. gondii* tachyzoite infection. DNA vaccination for the induction of both specific humoral and cellular immune responses against a lethal tachyzoite challenge has been studied in mice [31,32]. However, in comparison, PLG-rSAG1 microparticles prepared in the present study provide a much higher protection rate (80%) in BALB/c mice than the GRA6 DNA vaccine (53.3%) [32]. In addition, only survival prolongation (by 18 days), but no protection, has been observed in BALB/c mice immunized with another DNA vaccine, pSAG1/14-3-3 [31]. We therefore propose that PLG-rSAG1 microparticles may be superior to *Toxoplasma* DNA vaccines in protecting mice from the acute tachyzoite infection. However, due to critical influences of the parasite genotypes (types 1, 2 and 3) [33] and host susceptibility [34] on disease progression and severity of toxoplasmosis, the protective efficacy of *Toxoplasma* vaccines should be evaluated with different *T. gondii* strains and different host models. Further studies are therefore needed to confirm whether administration with PLG-rSAG1 microparticles protects different animal models from an oral challenge of *T. gondii* cysts (types 2 and 3), which imitates the natural infection initiated by ingesting oocysts released in cat faeces or consuming meat from infected animals containing the long-lived tissue cysts [1,6].

## Conclusions

We have successfully encapsulated rSAG1 with PLG polymers to produce PLG-rSAG1 microparticles that can sustain the stable release of antigenic rSAG1 for a long time (35 days). In addition, PLG-rSAG1 microparticles administered in the mouse peritoneal cavity enhance and maintain protective SAG1-specific humoral and cell-mediated immune responses for an extended period (10 weeks) to protect mice from *T. gondii* tachyzoite infection. The capability of this microparticle vaccine to control the stable release of antigenic rSAG1 and effectively induce and extend protective immunity would be advantageous for its application in the development of long-lasting vaccines against *T. gondii* for future use in humans and animals.

## Competing interests

The authors declare that they have no competing interests.

## Authors’ contributions

SCC carried out rSAG1 production, performed PLG encapsulation and drafted the manuscript. JCK performed microparticle assays including scanning electron microscopy and *in vitro* release. CPC and JTD participated in mouse immunization and challenge. CDY designed and coordinated the study and wrote the manuscript. All authors read and approved the final manuscript.

## References

[B1] Hill D Dubey JP Toxoplasma gondii: transmission, diagnosis and prevention Clin Microbiol Infect 2002 8 10 634 640 10.1046/j.1469-0691.2002.00485.x 12390281

[B2] Dubey JP The history of Toxoplasma gondii–the first 100 years J Eukaryot Microbiol 2008 55 6 467 475 10.1111/j.1550-7408.2008.00345.x 19120791

[B3] Kravetz JD Federman DG Toxoplasmosis in pregnancy Am J Med 2005 118 3 212 216 10.1016/j.amjmed.2004.08.023 15745715

[B4] Contini C Clinical and diagnostic management of toxoplasmosis in the immunocompromised patient Parassitologia 2008 50 1–2 45 50 18693556

[B5] Buxton D Toxoplasmosis: the first commercial vaccine Parasitol Today 1993 9 9 335 337 10.1016/0169-4758(93)90236-9 15463799

[B6] Bhopale GM Development of a vaccine for toxoplasmosis: current status Microbes Infect 2003 5 5 457 462 10.1016/S1286-4579(03)00048-0 12738002

[B7] Jongert E Roberts CW Gargano N Forster-Waldl E Petersen E Vaccines against Toxoplasma gondii: challenges and opportunities Mem Inst Oswaldo Cruz 2009 104 2 252 266 10.1590/S0074-02762009000200019 19430651

[B8] Innes EA Bartley PM Maley S Katzer F Buxton D Veterinary vaccines against Toxoplasma gondii Mem Inst Oswaldo Cruz 2009 104 2 246 251 19430650 10.1590/s0074-02762009000200018

[B9] Grimwood J Smith JE Toxoplasma gondii: the role of parasite surface and secreted proteins in host cell invasion Int J Parasitol 1996 26 2 169 173 10.1016/0020-7519(95)00103-4 8690540

[B10] Yang CD Chang GN Chao D Protective immunity against Toxoplasma gondii in mice induced by a chimeric protein rSAG1/2 Parasitol Res 2004 92 1 58 64 10.1007/s00436-003-0992-5 14605877

[B11] Jongert E Lemiere A Van Ginderachter J De Craeye S Huygen K D'Souza S Functional characterization of in vivo effector CD4(+) and CD8(+) T cell responses in acute Toxoplasmosis: an interplay of IFN-gamma and cytolytic T cells Vaccine 2010 28 13 2556 2564 10.1016/j.vaccine.2010.01.031 20117266

[B12] Suzuki Y Orellana MA Schreiber RD Remington JS Interferon-gamma: the major mediator of resistance against Toxoplasma gondii Science 1988 240 4851 516 518 10.1126/science.3128869 3128869

[B13] Jain S O'Hagan DT Singh M The long-term potential of biodegradable poly (lactide-co-glycolide) microparticles as the next-generation vaccine adjuvant Expert Rev Vaccines 2011 10 12 1731 1742 10.1586/erv.11.126 22085176

[B14] Singh M O'Hagan DT Recent advances in veterinary vaccine adjuvants Int J Parasitol 2003 33 5–6 469 478 12782048 10.1016/s0020-7519(03)00053-5

[B15] Sinha VR Trehan A Biodegradable microspheres for protein delivery J Control Release 2003 90 3 261 280 10.1016/S0168-3659(03)00194-9 12880694

[B16] Lim TY Poh CK Wang W Poly (lactic-co-glycolic acid) as a controlled release delivery device J Mater Sci Mater Med 2009 20 8 1669 1675 10.1007/s10856-009-3727-z 19283453

[B17] Newman KD Elamanchili P Kwon GS Samuel J Uptake of poly (D, L-lactic-co-glycolic acid) microspheres by antigen-presenting cells in vivo J Biomed Mater Res 2002 60 3 480 486 10.1002/jbm.10019 11920673

[B18] Heegaard PM Dedieu L Johnson N Le Potier MF Mockey M Mutinelli F Vahlenkamp T Vascellari M Sorensen NS Adjuvants and delivery systems in veterinary vaccinology: current state and future developments Arch Virol 2011 156 2 183 202 10.1007/s00705-010-0863-1 21170730

[B19] Stanley AC Buxton D Innes EA Huntley JF Intranasal immunisation with Toxoplasma gondii tachyzoite antigen encapsulated into PLG microspheres induces humoral and cell-mediated immunity in sheep Vaccine 2004 22 29–30 3929 3941 15364441 10.1016/j.vaccine.2004.04.022

[B20] Yang CD Chang GN Chao D Protective immunity against Toxoplasma gondii in mice induced by the SAG2 internal image of anti-idiotype antibody Parasitol Res 2003 91 6 452 457 10.1007/s00436-003-1006-3 14564510

[B21] Ghaderi R Carlfors J Biological activity of lysozyme after entrapment in poly(d, l-lactide-co-glycolide)-microspheres Pharm Res 1997 14 11 1556 1562 10.1023/A:1012122200381 9434274

[B22] Jeffery H Davis SS O'Hagan DT The preparation and characterization of poly (lactide-co-glycolide) microparticles. II. The entrapment of a model protein using a (water-in-oil)-in-water emulsion solvent evaporation technique Pharm Res 1993 10 3 362 368 10.1023/A:1018980020506 8464808

[B23] Byrd W Cassels FJ Intranasal immunization of BALB/c mice with enterotoxigenic Escherichia coli colonization factor CS6 encapsulated in biodegradable poly (DL-lactide-co-glycolide) microspheres Vaccine 2006 24 9 1359 1366 10.1016/j.vaccine.2005.09.024 16233937

[B24] Yang CD Liao JT Lai CY Jong MH Liang CM Lin YL Lin NS Hsu YH Liang SM Induction of protective immunity in swine by recombinant bamboo mosaic virus expressing foot-and-mouth disease virus epitopes BMC Biotechnol 2007 7 62 10.1186/1472-6750-7-62 17900346 PMC2117009

[B25] Subauste CS Remington JS Immunity to Toxoplasma gondii Curr Opin Immunol 1993 5 4 532 537 10.1016/0952-7915(93)90034-P 8216929

[B26] Casciotti L Ely KH Williams ME Khan IA CD8(+)-T-cell immunity against Toxoplasma gondii can be induced but not maintained in mice lacking conventional CD4(+) T cells Infect Immun 2002 70 2 434 443 10.1128/IAI.70.2.434-443.2002 11796568 PMC127655

[B27] Luzardo-Alvarez A Blarer N Peter K Romero JF Reymond C Corradin G Gander B Biodegradable microspheres alone do not stimulate murine macrophages in vitro, but prolong antigen presentation by macrophages in vitro and stimulate a solid immune response in mice J Control Release 2005 109 1–3 62 76 16269200 10.1016/j.jconrel.2005.09.015

[B28] Men Y Audran R Thomasin C Eberl G Demotz S Merkle HP Gander B Corradin G MHC class I- and class II-restricted processing and presentation of microencapsulated antigens Vaccine 1999 17 9–10 1047 1056 10195614 10.1016/s0264-410x(98)00321-1

[B29] Kang H Remington JS Suzuki Y Decreased resistance of B cell-deficient mice to infection with Toxoplasma gondii despite unimpaired expression of IFN-gamma, TNF-alpha, and inducible nitric oxide synthase J Immunol 2000 164 5 2629 2634 10679102 10.4049/jimmunol.164.5.2629

[B30] Johnson LL Sayles PC Deficient humoral responses underlie susceptibility to Toxoplasma gondii in CD4-deficient mice Infect Immun 2002 70 1 185 191 10.1128/IAI.70.1.185-191.2002 11748181 PMC127596

[B31] Meng M He S Zhao G Bai Y Zhou H Cong H Lu G Zhao Q Zhu XQ Evaluation of protective immune responses induced by DNA vaccines encoding Toxoplasma gondii surface antigen 1 (SAG1) and 14-3-3 protein in BALB/c mice Parasit Vectors 2012 5 1 273 10.1186/1756-3305-5-273 23181694 PMC3547689

[B32] Sun XM Zou J AE A Yan WC Liu XY Suo X Wang H Chen QJ DNA vaccination with a gene encoding Toxoplasma gondii GRA6 induces partial protection against toxoplasmosis in BALB/c mice Parasit Vectors 2011 4 213 10.1186/1756-3305-4-213 22070984 PMC3229464

[B33] Rajendran C Su C Dubey JP Molecular genotyping of Toxoplasma gondii from Central and South America revealed high diversity within and between populations Infect Genet Evol 2012 12 2 359 368 10.1016/j.meegid.2011.12.010 22226702

[B34] Li Z Zhao ZJ Zhu XQ Ren QS Nie FF Gao JM Gao XJ Yang TB Zhou WL Shen JL Differences in iNOS and arginase expression and activity in the macrophages of rats are responsible for the resistance against T. gondii infection PLoS One 2012 7 4 e35834 10.1371/journal.pone.0035834 22558235 PMC3338469

